# Community structure and carbon metabolism functions of bacterioplankton in the Guangdong coastal zone

**DOI:** 10.1007/s42995-024-00245-x

**Published:** 2024-07-29

**Authors:** Ziqi Peng, Pandeng Wang, Xiaoqing Luo, Qiqi Deng, Ziwen Yang, Jiaxue Wu, Wendong Xian, Weicong Yan, Xiaozhen Mou, Yang Yuan, Wenjun Li, Jialing Li

**Affiliations:** 1 State Key Laboratory of Biocontrol, Guangdong Provincial Key Laboratory of Plant Stress Biology, School of Marine Sciences, School of Ecology, School of Life Sciences, Sun Yat-Sen University, Guangzhou, 510275 China; 2 Southern Marine Science and Engineering Guangdong Laboratory (Guangzhou), Guangzhou, 511458 China; 3 Department of Biological Sciences, Kent State University, Kent, OH 44242 USA; 4 Marine Microorganism Ecological & Application Lab, Marine Science and Technology College, Zhejiang Ocean University, Zhoushan, 316000 China

**Keywords:** Particle-attached and free-living bacterioplankton, Community structure, Carbon metabolism, Coastal ecosystem

## Abstract

Coastal ecosystems are an important region for biogeochemical cycling, are a hotspot of anthropogenic disturbance and play a crucial role in global carbon cycling through the metabolic activities of bacterioplankton. Bacterioplankton can be broadly classified into two lifestyles: free-living (FL) and particle-attached (PA). However, how coastal bacterioplankton the community structure, co-occurrence networks and carbon metabolic functions with different lifestyles are differentiated is still largely unknown. Understanding these processes is necessary to better determine the contributions of coastal bacterioplankton to carbon cycling. Here, the characteristics of community structure and carbon metabolism function of bacterioplankton with two lifestyles in the coastal areas of Guangdong Province were investigated using amplicon sequencing, metagenomic, and metatranscriptomic techniques. The results show that the main bacterioplankton responsible for carbon metabolism were the *Pseudomonadota*, *Bacteroidota*, and *Actinomycetota*. The microbial community structure, carbon metabolic function, and environmental preferences differ between different lifestyles. FL and PA bacteria exhibited higher carbon fixation and degradation potentials, respectively. A range of environmental factors, such as dissolved oxygen, pH, and temperature, were associated with the community structure and carbon metabolic functions of the bacterioplankton. Human activities, such as nutrient discharge, may affect the distribution of functional genes and enhance the carbon degradation functions of bacterioplankton. In conclusion, this study increased the understanding of the role of microorganisms in regulating carbon export in coastal ecosystems with intense human activity.

**Supplementary Information:**

The online version contains supplementary material available at 10.1007/s42995-024-00245-x.

## Introduction

The ocean, as the largest carbon reservoir on earth, plays a crucial role in the carbon cycle and can buffer the impacts of climate change (Dithugoe Choaro et al. 2023). Primary production in surface seawater and the fixed carbon export to the deep sea are considered primary contributors to natural carbon sequestration (Schlitzer 2002; Siegenthaler and Sarmiento 1993). Bacterioplankton are a key component of marine ecosystems, actively participating in the biological (BCP) and microbial carbon pumps (MCP) through carbon fixation and degradation, and affecting the absorption and release of CO_2_ (Jiao et al. 2010). For instance, picocyanobacteria, represented by *Prochlorococcus* and *Synechococcus*, are the most abundant prokaryotic photoautotrophs on Earth. Through photosynthesis, they convert CO_2_ into organic carbon, contributing approximately 25% of the net marine primary productivity (Flombaum et al. 2013). Ideally, carbon fixed by these phytoplankton in surface water sinks and is buried as sediment, contributing to carbon storage for atmospheric CO_2_. However, the fixed carbon may subsequently be affected by microbial carbon degradation processes which can lead to remineralization and release during the sinking process. As a result, only a small portion of the fixed carbon reaches the bottom of the ocean (Giering et al. 2014; Martin et al. 1987), affecting the efficiency of carbon export (Azam et al. 1991; Fenchel 2008).

Bacterioplankton can be classified into two lifestyles: free-living (FL) and particle-attached (PA). In recent years, a number of studies have compared the differences between FL and PA microorganisms in terms of community structure, physiological characteristics, and metabolic functions (Crump et al. 1999; DeLong et al. 1993; Ganesh et al. 2014; Li et al. 2018). PA bacteria often exhibit higher diversity, being primarily composed of complex biopolymers/carbohydrates degrading taxa. They contain more genes associated with nutrient cycling, such as carbon degradation, nitrogen fixation, and polyphosphate degradation (Jain et al. 2019; Liu et al. 2020; Smith et al. 2013). However, the functional characteristics and differences in specific carbon metabolism processes between the two bacterial communities remain unclear, resulting in the differing influence of these two communities on carbon export process, especially in the highly human-disturbed coastal areas, being undefined. Coastal regions are one of the most important sites for the cycling and transformation of essential elements such as carbon and nitrogen (Djurhuus et al. 2020). Investigating the influence of bacterioplankton and human activities on carbon export process in this habitat may lead to a better understanding of the carbon sequestration function of marine ecosystems.

In recent years, rapid industrialization and urbanization have resulted in an influx of a large quantity of inorganic and organic particulates into coastal areas of Guangdong, leading to severe water eutrophication. The ecosystem and biogeochemical cycles in the coastal area of Guangdong Province are being strongly disturbed by pollutants (Duan et al. 2022; Strokal et al. 2015). Therefore, it is essential to identify environmental effects on microorganisms in this region and the ecological functions performed by them. To deepen the understanding of microbial communities in the coastal areas of Guangdong, water samples from 22 sites along the coastline were collected, and high-throughput amplicon sequencing, metagenomic, and metatranscriptomic techniques were used to determine the diversity, community structure, network properties, community assembly processes, environmental drivers, and carbon metabolism functions between the two different lifestyles. This study aims to enhance the knowledge of the ecological functions of bacterioplankton communities by investigating and analyzing different bacterial lifestyles, providing scientific data and a theoretical reference for the study of carbon cycling functions in coastal Guangdong.

## Materials and methods

### Study area and sampling strategy

**Fig. 1:**
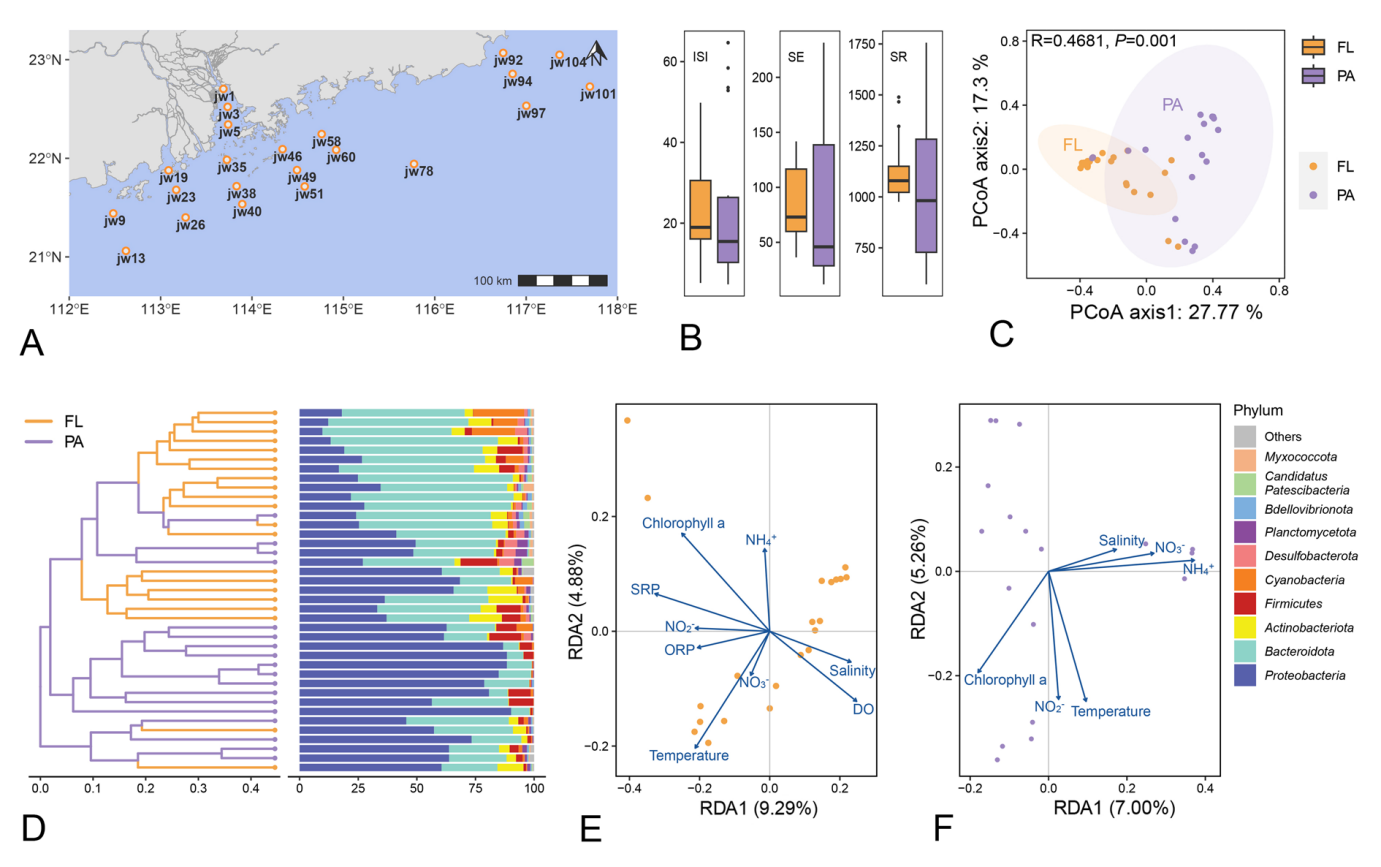
Overview of microbial community in Guangdong coastal zone. **A** Geographical location of sampling sites in Guangdong coastal zone. **B** Alpha diversity of different lifestyles. The free-living (FL) and particle-attached (PA) are denoted as orange and purple correspondingly. Abbreviation of alpha diversity indices: ISI: Inverse Simpson Index; SE: Shannon Entropy; SR: Species Richness. **C** Principal Coordinates Analysis (PCoA) plot, including all samples of the two lifestyles. **D** Cluster trees based on Bray–Curtis dissimilarity among all samples aligned with the relative abundance of different phyla. Top 10 abundant phyla were showed and the rest were incorporated and exhibited as “Others”. Redundancy Analysis (RDA) results of FL **E** and PA **F** microbial community

A field survey was conducted in June 2019, at the interface between the Pearl River Estuary and the shallow continental shelf of the South China Sea. A total of 22 sampling sites were set up on the coastline of Guangdong Province. These sites covered coastal regions, including Jiangmen, Zhuhai, Shenzhen, Huizhou, Shanwei, Jieyang, and Shantou, that are significantly impacted by human activities (Fig. 1A, Table S1). Seawater samples were collected at a depth of 1 m. Environmental parameters of the seawater were measured in situ using a Conductivity Temperature Depth (CTD, Sea-Bird Scientific) profiler. These included temperature, dissolved oxygen (DO), Chlorophyll a (Chl a), salinity, and pH. Immediately after collecting, each of the water samples was filtered through a 3 μm filter (Isopore™, 142 mm, Millipore) and a 0.2 μm filter (Supor-200, 142 mm, Pall Life Sciences) to collect PA and FL microbial communities, respectively. The 44 membrane filters (2 from each sampling site) were then transferred to sterile plastic bags and frozen in liquid nitrogen before being stored in a –80 °C freezer in the laboratory. The filtered water samples were stored in sterile centrifuge tubes at 4 °C for further chemical analysis.

### Biochemical properties, DNA and RNA extraction, and sequencing

Dissolved organic carbon (DOC) analysis was performed using a TOC analyzer (TOC-vcph; Shimadzu Corp., Tokyo, Japan) following standard methods. The concentrations of nitrate (NO_3_^−^), nitrite (NO_2_^−^), ammonium (NH_4_^+^), dissolved nitrogen (DN), and soluble reactive phosphorus (SRP) were determined using a fully automated intelligent chemical analyzer (SmartChem; Westoco Scientific Instruments Inc., Brookfield, CT, USA).

Total DNA and RNA were extracted from each of the 44 filter samples (22 sites × 2 lifestyles) using RNeasy PowerSoil Total RNA Kit (QIAGEN, Germany) according to the manufacturer’s protocol. The DNA and RNA were separately eluted from the capture column by RNeasy PowerSoil DNA Elution Kit (QIAGEN) and RNeasy PowerSoil Total RNA Kit (QIAGEN). DNA and RNA samples were then sent to the Guangdong Magigene Biotechnology Company for amplicon, metagenomic and metatranscriptomic sequencing. The V4 and V5 hypervariable regions of the bacterial 16S rRNA gene were amplified using forward primer 515F (5′-GTGYCAGCMGCCGCGGTAA-3′) and reverse primer 926R (5′-CCGYCAATTYMTTTRAGTTT-3′) (Parada et al. 2016). Amplicon sequencing was performed on the Illumina NovaSeq 6000 PE250 platform. The metagenomic and metatranscriptomic sequencing were performed on the Illumina Hiseq PE150 platform.

### Microbial community analysis

The amplicon sequencing data were further analyzed following the USEARCH pipeline (Edgar 2010); after merging paired-end reads, primer sequences were removed using CUTADAPT v2.4 (Martin 2011). Subsequently, sequences with a maximum expected error greater than 1.0 or a length less than 350 bp were filtered out. The remaining high-quality reads were dereplicated using the unoise3 algorithm, clustering sequences into amplicon sequence variants (ASVs) at a 100% similarity threshold. Taxonomic annotation of ASVs was performed using the Naïve Bayes classifier, based on the SILVA 132 database (Quast et al. 2012) on the QIIME2 platform (qiime2-2022.11) (Bolyen et al. 2019). ASVs that were not annotated as bacteria or archaea were removed (Kaehler et al. 2019). The reads, with primers removed, were then mapped back to ASV representative sequences to generate an ASV table. To correct for the impact of sequencing depth, the ASV table was rarefied to the lowest number of reads across all samples (46,403), resulting in a final dataset of 5,262,136 high-quality sequences. ASVs along with their frequency distribution and taxonomic annotation information were used for downstream statistical and diversity analysis.

Alpha diversity indices were used to measure the diversity within individual samples, reflecting microbial community abundance and diversity. Bray–Curtis distance was calculated to assess differences between samples, followed by Principal Coordinate Analysis (PCoA) for visualization. The similarity analysis (ANOSIM) in the vegan package was used to determine if the differences between groups were significantly different from the differences within group.

Redundancy Analysis (RDA) was employed to establish links between changes in microbial community composition and the corresponding environmental variables. Before RDA, Variance Inflation Factors (VIFs) were calculated for each environmental variable to identify collinearity. Variables with the highest VIFs were removed until all variables had a VIF below 10 (Zhang et al. 2021). Next, the Bioenv function from the vegan package was used to further select combinations of environmental factors that exhibited strong correlations with community differences. The first two canonical axes of the RDA results were visualized to show sites and explanatory variables. Additionally, the Hierarchical Partitioning (HP) method was used to assess the contribution of environmental factors to changes in community structure. This step utilized the rdacca.hp function from the rdacca.hp package (Lai et al. 2022).

### Co-occurrence network analysis

The ASV table was taxonomically annotated to obtain abundance charts of bacterial communities at different taxonomic levels. Subsequently, to enhance the robustness of network construction, rare ASVs, i.e., those present in less than one-third of the samples, were filtered out. SparCC software was then employed to infer correlations between species within the two microbial community types, constructing a microbial species interaction network (Friedman and Alm 2012). Based on the constructed network, several topologic characteristics were calculated. To determine the ecological roles of each node, two topological properties were considered: within-module degree (*Zi*) and among-module connectivity (*Pi*), indicating how ‘well connected’ a node is to nodes within their own module or from other modules, respectively (Guimerà and Amaral 2005). Specifically, based on their *Zi* and *Pi* values, all nodes were assigned to one of four roles: module hubs (*Zi* > 0.25, *Pi* ≤ 0.62), connectors (*Zi* ≤ 0.25, *Pi* > 0.62), network hubs (*Zi* > 0.25, *Pi* > 0.62), and peripherals (*Zi* ≤ 0.25, *Pi* < 0.62) (Olesen et al. 2007). Nodes other than peripheral nodes in each network were defined as potential ecologically important key species (Zhang et al. 2021). The network was visualized using Gephi (0.10.1) (Bastian et al. 2009).

### Quantification of microbial community assembly processes

In this study, the relative importance of different community assembly processes was quantified using the null-model approach described by Stegen et al. (2013). The null-model approach distinguishes between selection processes and neutral processes by calculating standardized phylogenetic turnover (βNTI) between communities. The βNTI of FL and PA were calculated, respectively, using the iCAMP package in R. βNTI less than -2 or greater than 2 indicate homogeneous selection or heterogeneous selection, respectively. |βNTI|< 2 suggests neutral processes. Subsequently, the Bray–Curtis-based Raup–Crick metric (RCbray) was used to further categorize specific neutral processes, with RCbray < -0.95 inferring homogeneous dispersal, RCbray > 0.95 representing dispersal limitation, and |RCbray|≤ 0.95 indicating drift (Osburn et al. 2021; Stegen et al. 2013, 2015).

### Identification and calculation of carbon metabolism genes

The raw metagenomic reads were trimmed using fastp (Chen et al. 2018) (v0.20.0, parameters: -n 0 -l 30 –cut_front –cut_right –cut_window_size 4 –cut_mean_quality 30). High-quality metagenomic reads for each sample were then assembled using MEGAHIT v1.2.9 (Li et al. 2016) with parameters '–k-min 27 –k-max 127 –k-step 20 –min-contig-len 500’. Open-reading frames (ORFs) were predicted from all contigs using Prodigal v2.6.3 (Hyatt et al. 2010) with parameters ‘-m -p meta’.

A reference database related to carbon metabolism-related genes was developed using amino acid (aa) sequences manually downloaded from the Nr database, according to previous studies (Shi et al. 2019; Tu et al. 2014), which is mainly consisted of seven common carbon sources in natural carbon degradation processes (Table S2) and six microbial carbon fixation processes (Table S3). Then, the ORFs were searched against the reference database using DIAMOND v0.9 (Buchfink et al. 2015) with parameters ‘-k 1 –evalue 0.00001 –id 50 –query-cover 80’. To construct a non-redundant gene catalog (467,177 ORFs), the results after annotation were clustered at a 95% similarity level (Li et al. 2014; Sunagawa et al. 2015) using CD-HIT-EST v4.8.1 (Li and Godzik 2006) with parameters ‘-c 0.95 -n 10 -G 0 -aS 0.9 -g 1 -d 0 -T 15 -M 0’ (Bahram et al. 2018). The abundance of each ORF across samples was calculated by mapping metagenomic high-quality reads to the gene catalog using BBMap (Bushnell 2014) with parameters ‘minid = 0.95’. In this study, Fragments Per Kilobase of exon model per Million mapped fragments (FPKM) was used to measure the relative abundance of mapped genes related to carbon degradation and carbon fixation.

The quality of raw metatranscriptomic reads was also controlled by fastp (Chen et al. 2018) (v0.20.0, parameters: -n 0 -l 30 –cut_front –cut_right –cut_window_size 4 –cut_mean_quality 30). Before further analysis, RiboDetector was used to clear ribosomal RNA. Then, the transcript abundance of each ORF was calculated by mapping high-quality metatranscriptomic reads to the gene catalog using BBMap (Bushnell 2014) with parameters ‘minid = 0.95’. Similarly, the relative transcript abundance of mapped genes related to carbon degradation and fixation was also shown by FPKM.

### Taxonomy assignment of functional genes

To determine the taxonomy of the carbon metabolism genes, they were searched against the GTDB r214 protein database (https://gtdb.ecogenomic.org/) by mmseqs easy-taxonomy (Mirdita et al. 2021) with default parameters. The contribution of different phyla to each carbon metabolism-related gene was calculated as the sum of the abundances of sequences that were assigned to that phylum.

### Genome binning, curation, dereplication, and annotation

Metagenome-Assembled Genomes (MAGs) were recovered from each sample using previously described methods (Luo et al. 2022; Wang et al. 2022). Briefly, scaffolds of each sample were binned by MetaBAT2, based on the coverage variation of contigs across samples and tetranucleotide frequencies with default parameters (Kang et al. 2019). The qualities of MAGs were evaluated using CheckM v1.0.12 (Parks et al. 2015), and then, the potential contaminations of each MAG were identified and removed by RefineM v0.0.25 (Parks et al. 2017) and manual curation. MAGs with high-to-medium-quality (completeness > 60%, contamination < 5%) from all samples were combined and dereplicated using dRep v2.6.2 (Olm et al. 2017) with default settings. The taxonomy of each MAG was determined by GTDB-Tk v1.2.0 (Chaumeil et al. 2019). ORFs in MAGs were predicted using Prodigal v2.6.3 (Hyatt et al. 2010) with default parameters. Then, the ORFs were searched against the reference database by DIAMOND v0.9 (Buchfink et al. 2015) with parameters as described in Sect. “Identification and calculation of carbon metabolism genes” to construct a gene catalog (7865 ORFs). Gene abundance and transcript abundance of carbon metabolism genes in each MAG were quantified as described in Sect. “Identification and calculation of carbon metabolism genes”. The abundance of each MAG in every sample was estimated based on the FPKM values of its constituent scaffolds.

### Statistical analysis

All statistical analyses were performed using R software (version 4.3.1). The significance of differences in α-diversity, genomic abundance, and transcriptomic abundance of carbon metabolism genes between FL and PA were determined using Wilcoxon test. The relationship between relative abundance of carbon metabolism genes and environmental traits was analyzed based on Pearson's correlation method. The spatial distributions of abundance of carbon metabolism genes were interpolated across the PRE using the IDW method in GSTAT package (Gräler et al. 2016).

## Results

### Structure, environmental drivers, and assembly process of bacterioplankton community with different lifestyles

Amplicon data from 39 filter samples were successfully obtained. There was no significant difference in α-diversity between FL and PA communities (*P* > 0.05, Fig. 1B). The result of principal coordinate analysis (PCoA) indicates significant differences (ANOSIM, R = 0.4681, *P* < 0.05) in community structure between the FL and PA groups (Fig. 1C).

A total of 19 phyla and 60 classes were detected. *Pseudomonadota* and *Bacteroidota* were the most common phyla in each community (ranging from 64.8% to 99.0%). The FL community was mainly composed of *Bacteroidota*, while the PA community was mainly composed of *Pseudomonadota* (Fig. 1D). Additionally, the microbial composition in each sample was clustered based on their Bray–Curtis distances and, except for a few samples, FL and PA clustered separately (Fig. 1D).

To identify the environmental variables influencing the community structure of FL and PA, RDA was performed separately for FL and PA. After filtering based on VIF and BioEnv analysis, nine and six environmental variables were retained for RDA analysis in FL and PA groups, respectively. In the FL group, temperature, DO, and Chl a were identified as the primary factors shaping microbial community structure (Fig. 1E), with temperature exerting the most significant impact (6.9%, *P* < 0.05). In the PA group, NH_4_^+^, temperature, and NO_3_^−^ were the main environmental drivers (Fig. 1F), with NH_4_^+^ being the most important driver (7.3%, *P* < 0.05).

Stochastic processes were the primary assembly mechanisms for the two bacterial communities (Fig. S1A). The role of dispersal limitation was important for bacterial community assembly in both the FL (51.9%) and PA (51.6%) community (Fig. S1B). Additionally, heterogeneous selection processes constituted a significant proportion in FL (36.7%) and PA (34.6%), indicating microbial adaptability to diverse environmental conditions in the Guangdong coastal zone (Fig. S1B).

### Co-occurrence networks with different lifestyles

**Fig. 2:**
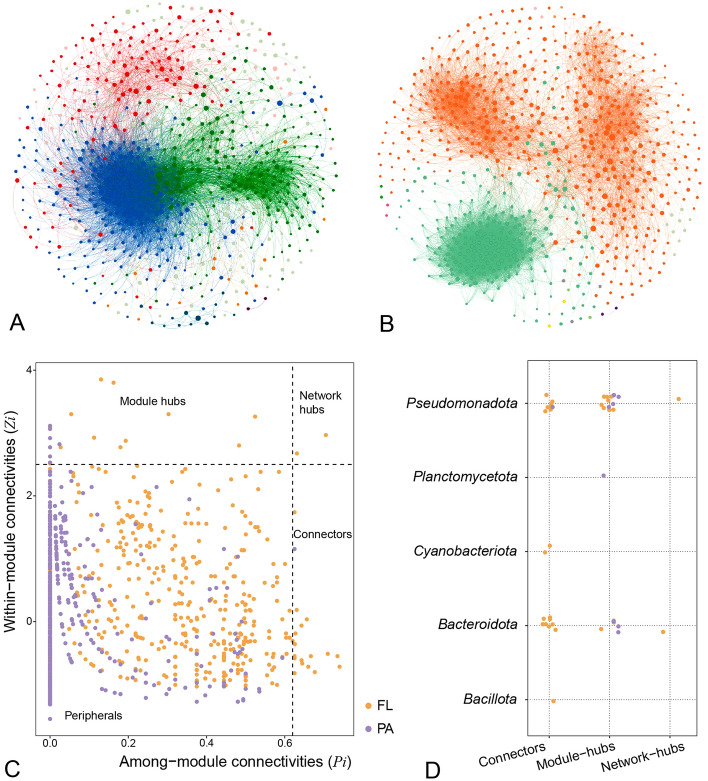
The molecular ecological networks of the free-living (FL) and particle-attached (PA) microbial communities. **A** FL network. **B** PA network. The color of the nodes indicates different modules and the size is proportional to log_2_(ASV numbers). **C** The different ecological role of all nodes, partitioned at *Zi* = 2.5 and *Pi* = 0.62. The nodes of free-living (FL) and particle-attached (PA) are denoted as orange and purple correspondingly. **D** The taxonomy of connectors, module hubs and network hubs at phylum level

Microbial interaction networks for FL and PA were constructed using the SparCC method (Fig. 2A, B). These networks exhibited a range of topological properties. Compared to FL, the PA network had a higher number of nodes and connections, indicating a more complex network structure (Table 1). Additionally, the PA network exhibited higher values for average degree (AD), graph density (GD), and clustering coefficient (CC), suggesting that the bacterial community structure in PA was more tightly interconnected than in FL.

**Table 1:** Topological properties of the co-occurrence network in FL and PA

Lifestyle	FL	PA
Total nodes	724	737
Total links	9985	13,378
Average degree	27.58	36.3
Average path length	2.07	2.1
Graph diameter	5.94	5.65
Graph density	0.038	0.049
Clustering coefficient	0.52	0.66
Betweenness centralization	0.04	0.045
Degree centralization	0.187	0.165
Modularity	0.33	0.471

To identify potential keystone taxa in each network, each node was classified into four ecological roles based on their within-module degree (*Zi*) and among-module connectivity (*Pi*) (see methods, Fig. 2C). The PA community had nine keystone nodes, most of which served as module hubs critical for maintaining the stability of module structures. These keystone taxa belonged to *Pseudomonadota*, *Bacteroidota*, and *Planctomycetota* (Fig. 2D). In contrast, the FL community had 32 keystone nodes, which was significantly more than PA, comprising 12 module hubs, 18 connector nodes, and 2 network hubs. These nodes played essential ecological roles in maintaining the stability and functionality of the network. Similarly, they were primarily composed of bacteria from *Pseudomonadota* and *Bacteroidota* (Fig. 2D).

### Carbon metabolism functions of *bacteria* with different lifestyles

Metagenomic data from 38 samples and metatranscriptomic data from 21 samples were successfully obtained (Table S4). The gene abundance and transcriptional abundance in carbon degradation (CD) and carbon fixation (CF) processes were first compared between FL and PA. The results showed that PA had a higher abundance of carbon degradation genes, while FL had a higher abundance of carbon fixation genes (Fig. S2). This suggests that in this study, FL and PA may dominate different carbon cycling processes in coastal ecosystems.

**Fig. 3:**
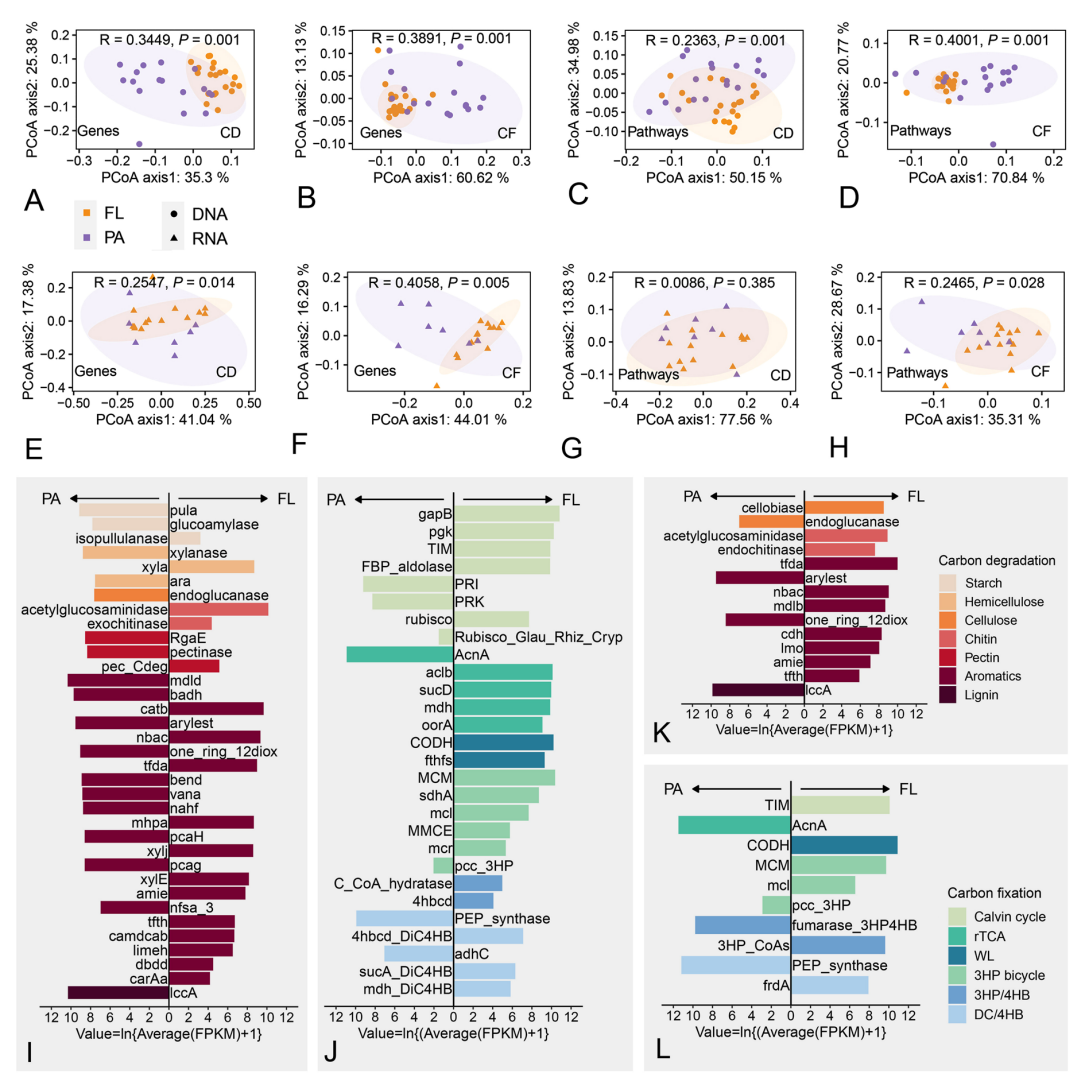
Differences in carbon degradation and carbon fixation functions between the free-living (FL) and particle-attached (PA) microbial communities. Principal coordinates analysis (PCoA) of carbon degradation (CD) based on genes **(A, E)** or pathways **(C, G)** and PCoA of carbon fixation (CF) based on genes **(B, F)** or pathways **(D, H)**. The circular dots represent metagenomic data **(A ~ D)** and the triangular dots represent metatranscriptomic data **(E ~ H)**. Fragments Per Kilobase Million (FPKM), after taking natural logarithm, were used to represent the abundance of carbon metabolism genes in metagenomes **(I, J)** and metatranscriptomes **(K, L)** of the sampling points. Wilcoxon rank-sum test of each gene was performed to examine whether the difference between FL and PA was significant. Only genes with significant differences were shown. Gene directed toward the arrow showed FL (or PA) means that there was more of that gene in FL (or PA). Abbreviation in carbon fixation process: rTCA cycle: reductive tricarboxylic acid cycle; WL: reductive acetyl-CoA pathway; 3HP bicycle: 3-hydroxypropionate bicycle; 3HP/4HB: 3-hydroxypropionate/4-hydroxybutyrate cycle; DC/4HB: dicarboxylate/4-hydroxybutyrate cycle

To investigate whether there were differences in specific carbon metabolism processes among bacterioplankton with different lifestyles, the composition (Fig. 3A–H) and abundance (Fig. 3I–L, Fig. S2) of genes involved in carbon degradation (Table S2) and carbon fixation (Table S3) in FL and PA were analyzed. Seven common carbon sources in natural carbon degradation processes and six microbial carbon fixation processes are listed in Fig. 3. A PCoA analysis at both the gene and metabolic pathway levels was conducted and the results showed that there were significant differences in carbon degradation and carbon fixation functions between FL and PA (ANOSIM, *P* < 0.05), suggesting that FL and PA exhibit distinct preferences for specific functional genes and metabolic pathways when performing carbon cycling. This phenomenon is further illustrated in the barplot in Fig. 3, where genes with significant differences in abundance in the genomes (Fig. 3I–J) and the transcriptome (Fig. 3K–L) between FL and PA are shown. These differences highlight the distinct carbon metabolism characteristics of the two kinds of bacteria with different lifestyles. For example, in terms of the degradation of aromatic hydrocarbons, FL had a higher variety and abundance of dioxygenase and monooxygenase genes (such as *nbac*, *xylE*, *carAa*, and *camdcab*), which played crucial roles in the cleavage of aromatic rings. PA, however, seemed to have the advantage in the side-chain modification process of aromatic hydrocarbons (such as *mdld*, *nfsa*, and *bend*). In terms of the six carbon fixation processes, there was a higher abundance of functional genes in FL, indicating that FL may have higher carbon fixation potential than PA in coastal surface seawater.

### Spatial distribution of carbon metabolism genes and their response to environmental factors

**Fig. 4:**
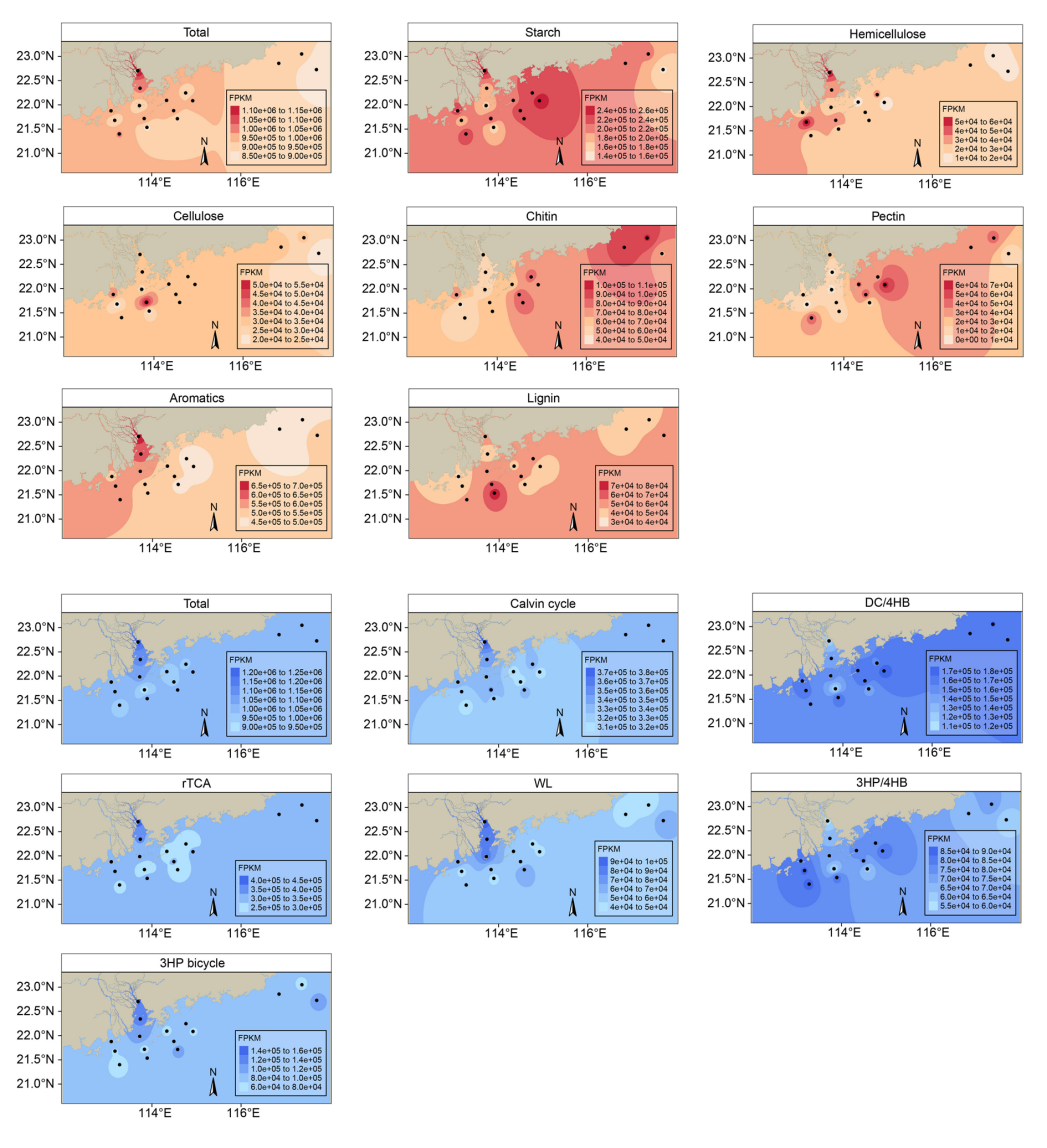
Spatial distribution of the abundance of carbon degradation and carbon fixation genes in metagenomes of bacterioplankton in Guangdong coastal zone

The genes contributing to the same carbon metabolism processes were combined and the distribution characteristics of carbon metabolism genes at different sampling sites are summarized (Fig. 4). Overall, both the carbon degradation and carbon fixation gene abundances were relatively higher in Pearl River Estuary, suggesting that estuaries may be hotspots for carbon cycling in coastal regions.

**Fig. 5:**
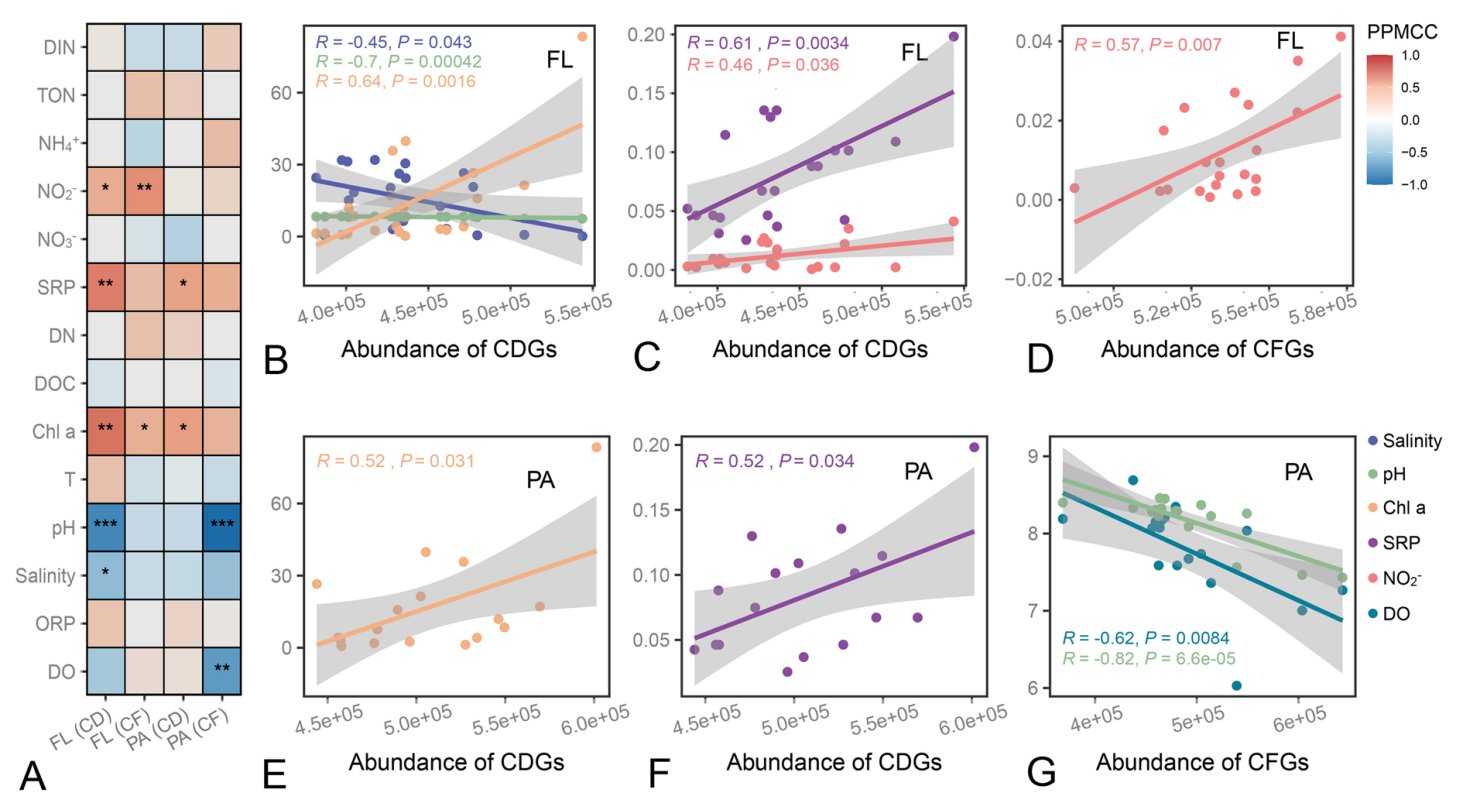
Correlation analysis between relative abundance of carbon metabolism genes and environmental traits. **A** Pearson correlation heatmap between abundance of carbon degradation genes (CDGs) and carbon fixation genes (CFGs) in FL and PA. The significance levels were denoted as “*”, 0.01 < *P* ≤ 0.05; “**”, 0.001 < *P* ≤ 0.01; “***”, *P* ≤ 0.001. **B** The relationship between CDGs abundance and salinity, pH or Chl a in FL bacteria. **C** The relationship between CDGs abundance and SRP or NO_2_^−^ in FL bacteria. **D** The relationship between CFGs abundance and NO_2_^−^ in FL bacteria. **E** The relationship between CDGs abundance and Chl a in PA bacteria. **F** The relationship between CDGs abundance and SRP in PA bacteria. **G** The relationship between CFGs abundance and pH or DO in PA bacteria. PPMCC: Pearson product–moment correlation coefficient

Additionally, the response of bacterioplankton in carbon metabolism functions to the physicochemical properties in coastal environment was explored, and the correlation between carbon metabolic gene abundance and environmental factors was investigated (Fig. 5). The results showed that six environmental factors can affect the carbon metabolic function of bacterioplankton, including pH, Chl a, salinity, DO (Fig. 5B, E, G), as well as nutrient-related factors such as NO_2_^−^ and SRP (Fig. 5C, D, F). Notably, as with community structure, the abundance of carbon metabolism genes in FL was associated with more environmental factors, suggesting that FL communities may be more sensitive to environmental changes.

### Relative contributions of community members to different processes

**Fig. 6:**
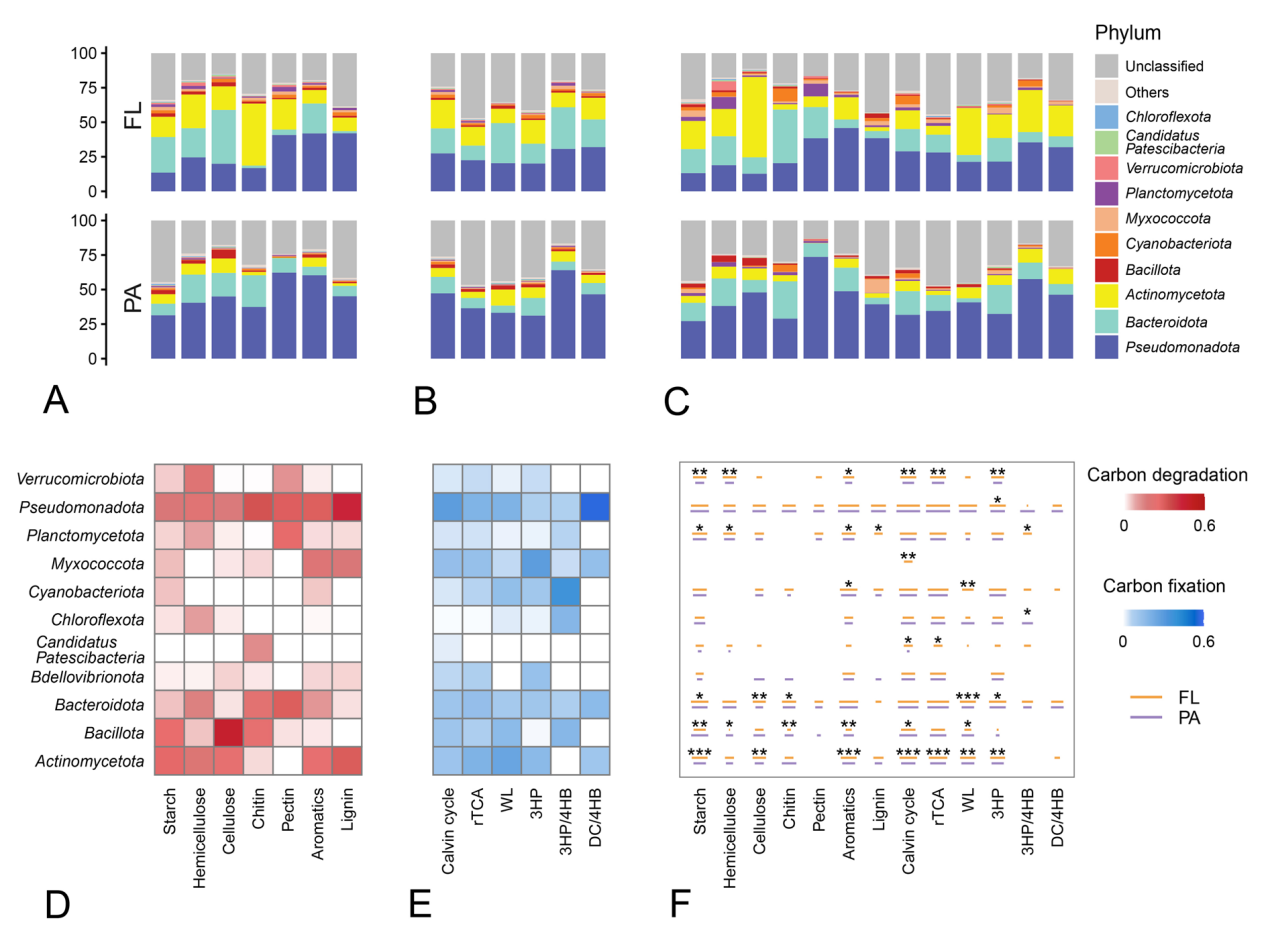
Relative contributions of different phyla to different carbon metabolism processes in Guangdong coastal zone. The relative contributions of different phyla to the gene abundances **(A, B)** and transcriptional abundance **(C)** of carbon metabolism-related genes. Average abundance of carbon degradation **(D)** and carbon fixation **(E)** related genes in MAGs from different phyla, expressed as a percentage of the total abundance across all phyla. **(F)** Transcriptional abundance of carbon metabolism-related genes in MAGs of different phyla with different lifestyles during each carbon cycle process. The length of the segments is proportional to ln (FPKM + 1). Wilcoxon rank-sum test was performed to examine whether the difference between FL and PA group is significant. The significance levels are denoted as “*”, 0.01 < *P* ≤ 0.05; “**”, 0.001 < *P* ≤ 0.01; “***”, *P* ≤ 0.001

To further explain the relationship between community members and functional capacities, the taxonomy of each functional gene was annotated. The results indicate that more genes were annotated as *Pseudomonadota* (32.6%), *Actinomycetota* (11.3%), and *Bacteroidota* (10.7%). Relative contributions of different phyla to certain carbon metabolism process were assessed based on the relative abundance of genes or transcripts from each phylum (Fig. 6A–C). Overall, in the PA community, *Pseudomonadota* had a notably higher gene abundance (Fig. 6A, B) and transcriptional activity (Fig. 6C). In contrast, in the FL community, the relative contribution of *Pseudomonadota* was comparatively lower, with a wider variety of phyla playing a significant role.

MAGs were recovered to identify the key taxa involved in carbon cycling functions within the coastal habitats of Guangdong. A total of 2793 prokaryotic genomes were assembled from 38 metagenomes. The MAGs were then refined and dereplicated into 332 representative genomes (Table S5). Most of the obtained MAGs belonged to *Pseudomonadota* (114/332), followed by *Bacteroidota* (110/332). Average abundance of carbon degradation (Fig. 6D) and carbon fixation (Fig. 6E) related genes, as well as transcriptional abundance (Fig. 6F) in MAGs from different phyla were calculated. Similarly, *Pseudomonadota* and *Bacteroidota* exhibited higher levels of carbon metabolism. The distinct characteristics shown by many taxa in the two lifestyles were further compared. Among *Pseudomonadota*, which demonstrated a significant role, the choice of lifestyle did not significantly affect its carbon metabolic capability. This may suggest that *Pseudomonadota* exhibited a wide and strong adaptability to various lifestyles. In contrast, in *Actinomycetota*, *Bacillota*, *Bacteroidota*, *Planctomycetota*, and *Verrucomicrobiota*, FL and PA displayed more inconsistencies, indicating that these phyla may have different selective preference for lifestyle.

## Discussion

This study showed that within the coastal regions of Guangdong, the primary bacterioplankton responsible for carbon metabolism were *Pseudomonadota*, *Bacteroidota*, and *Actinomycetota*. The selection of lifestyle significantly influenced the structure, ecological function, and environmental preferences of bacterioplankton communities, subsequently impacting the carbon cycling mechanisms within these coastal habitats. Overall, FL demonstrated a greater capacity for carbon fixation, while PA had a greater capacity for carbon degradation. When it comes to specific pathways of carbon metabolism, FL and PA communities performed better in different pathways, seeming to be able to complement each other. The distribution of their functional genes was influenced by a range of physicochemical factors and human activities.

### Distribution patterns of carbon metabolic functions in Guangdong coastal zone

In the coastal habitats of Guangdong, the primary phyla were *Pseudomonadota*, *Bacteroidota*, *Actinomycetota*, *Bacillota*, and *Cyanobacteriota*, which is consistent with studies from other relevant aquatic environments (Harrison et al. 2008). Additionally, as with the amplicon results, the annotation of functional genes and MAGs indicates that a broad and strong carbon cycle functional spectrum was distributed within *Pseudomonadota*, *Bacteroidota*, and *Actinomycetota* (Fig. 6). Similar results have been found in studies from the Southern Ocean, where *Pseudomonadota* and *Actinomycetota* had a greater number of genes involved in the degradation of carbohydrates, such as chitin and cellulose (Dithugoe Choaro et al. 2023). Notably, some low-abundance groups also played important roles in certain carbon metabolism processes in this study. For instance, *Myxococcota*, which was less abundant, lacked the degradation activities for hemicellulose and pectin, but possessed a relatively strong ability for aromatics degradation and Calvin cycle. Similarly, some studies have also emphasized the significant roles of *Myxococcota* in photosynthesis (Li et al. 2023).

In this study, the Pearl River Estuary showed greater vitality in carbon metabolism, with most of the carbon metabolic genes highly accumulated within the regions (Fig. 4). Some of carbon metabolic genes, however, were distributed with different patterns. For example, it was found that the abundance of chitin degradation genes was highest near Shanwei, Jieyang, and Shantou, which are distant from the estuary. This may have resulted from aquaculture activities, because these three coastal cities are known for mariculture, including chitin-rich crustaceans such as prawn (e.g., *Litopenaeus vannamei*) and crabs (e.g., *Scylla serrata*), which may contribute to the accumulation of chitin in the region and eventually led to the enrichment of genes related to chitin degradation.

### Community structure and functional characteristics of different lifestyles

In this study, particles had significant impact on the composition, co-occurrence patterns and carbon metabolism capability of bacterial communities. In the PA community, *Pseudomonadota* had a higher relative abundance compared to the FL community. Additionally, at the class level, *Gammaproteobacteria* was the most abundant taxon within the PA group (Fig. S3). This may result from the process of microbial colonization on particle surfaces, as previous research has suggested that *Gammaproteobacteria* are pioneering organisms in marine biofilm formation (Lee et al. 2008).

The co-occurrence network formed by PA bacteria was denser, with more distinct modules and more complex correlations within modules than FL bacteria. Similarly, many studies have shown that PA bacteria often have a more complex co-occurrence network structure compared to FL bacteria (Xu et al. 2018; Zhang et al. 2016). This suggests that particles serve as colonization points that make interactions such as cooperation and competition among bacteria more pronounced. Cooperation can help different PA bacteria collectively degrade the particles they colonized, while the limited space on particle surfaces may lead to strong competition for ecological niches among PA bacteria (Clauset et al. 2004; Xu et al. 2021). Despite the lower complexity of the FL network, the number of potential keystone species in the FL network exceeded that of the PA network, whose metabolic functions may be important in ecological services (Banerjee et al. 2018; Rafrafi et al. 2013). For example, two network hubs in the FL network, *Acinetobacter* and *Flavobacterium* were identified, both of which can degrade a range of organic pollutants, such as petroleum and polycyclic aromatic hydrocarbons (PAHs), suggesting the potential importance of FL communities in pollution remediation in the coastal areas of Guangdong (Chen et al. 2014; Liu et al. 2019). The key taxon found in both of the microbial networks was *Rhodobacteraceae*, which is mainly comprised of aerobic photo- and chemoheterotrophs but also purple non-sulfur bacteria, performing photosynthesis in anaerobic environments. It is deeply involved in sulfur and carbon biogeochemical cycling and symbiosis with micro- and macroorganisms in marine ecosystems (Dogs et al. 2017) and makes a significant contribution to biogeochemical processes.

The characteristics of planktonic bacterial carbon metabolic potentials in the surface water of the Guangdong coastal region were further investigated from a molecular aspect. First, carbon degradation and carbon fixation potential of FL and PA were broadly investigated. The results show that different lifestyle selection affected the carbon metabolic function of the bacterioplankton (Fig. 3A–H, with higher abundance of carbon degradation genes in PA and higher abundance of carbon fixation genes in FL, respectively (Fig. S2). This is consistent with the results of previous studies, in which PA were found to be more active in the degradation of particulate matter, while FL tended to be oligotrophic autotrophs (Herndl and Reinthaler 2013; Karner and Herndl 1992; Smith et al. 2013). In this study, the abundance of *Cyanobacteriota* in FL was higher, and *Synechococcus* and *Cyanobium* were the two key groups in the FL network; this further demonstrates the dominant position of the FL group in carbon fixation processes. The carbon metabolic genes that differed between FL and PA are further listed in Fig. 3I–L. These suggest that they may dominate different specific carbon metabolism processes and complement each other. For example, the degradation of cellulose requires the joint action of cellobiase and endoglucanase, and the transcription levels of genes encoding these two enzymes were more abundant in FL or PA, respectively (Fig. 3K).

### Environmental preference of different lifestyles

The microbial community structures of these two lifestyles were influenced by a range of environmental factors. Overall, temperature had a substantial impact on both the FL and PA community structures. Additionally, DO and NH_4_^+^ were major factors affecting the FL and PA communities, respectively (Fig. 1E–F). Similar results have been observed in the previous studies (Ma et al. 2023). Temperature can regulate bacterial metabolic capabilities by influencing enzyme activity, ultimately affecting bacterial community distribution and function. High DO is essential for aerobic metabolism in aquatic bacteria, with FL being more dependent on DO than PA (Aldunate et al. 2018; Mohiuddin et al. 2019). Studies have shown that organic particles in eutrophic water can absorb NH_4_^+^ (Lehmann et al. 2004), and the NH_4_^+^ adsorbed on particles affects the nitrogen cycle of PA, significantly influencing their community structure.

There was a negative correlation of pH and salinity with carbon degradation gene (CDG) abundance in FL groups (Fig. 5B). Similarly, pH exhibited a negative correlation with CFG abundance in PA groups (Fig. 5G). This might be because higher pH and salinity inhibited enzyme activity. It is worth noting that NO_2_^−^ and SRP, which can serve as N and P nutrients, were positively correlated with CDG abundance (Fig. 5C, F), seemingly promoting bacterial carbon degradation. Similar results have been observed in other habitats, including soil (Li et al. 2021a, 2021b), mangroves (Li et al. 2024), and estuaries (Yuan et al. 2010), where the input of N and P tended to stimulate microbial mineralization, which is detrimental to carbon storage. This suggests that achieving an increase in carbon sequestration requires a stronger control of nutrient inputs to the ocean (Jiao et al. 2011). Similarly, the concentration of Chl a can serve as an indicator of water eutrophication, which may be one of reasons why its effect on the abundance of CDGs was consistent with that of nutrients (Fig. 5B, E). Additionally, DO exhibited a negative correlation with CFG abundance in PA (Fig. 5G); this may be due to the fact that many processes among the six carbon fixation pathways are dependent on anaerobic conditions, such as the dicarboxylate/4-hydroxybutyrate cycle (DC/4HB), which is a strictly anaerobic carbon fixation process found in archaea (Huber et al. 2008).

## Conclusion

Utilizing amplicon, metagenomic and metatranscriptomic techniques, it was found that within the coastal regions of Guangdong, the primary bacterioplankton responsible for carbon metabolism were *Pseudomonadota*, *Bacteroidota*, and *Actinomycetota*. Different selection of lifestyle mode affected the structure, function, and environmental preference of bacterioplankton communities. FL and PA communities had stronger carbon fixation and carbon degradation potential, respectively, and thus made different contributions and had different influences on carbon storage processes in coastal ecosystems. Similar to previous studies, this study shows that nutrient input enhances the carbon degradation function of bacterioplankton, suggesting that nutrient input from land needs to be reduced. This would contribute to the realization of ocean negative carbon emissions (ONCE).

### Supplementary Information

Below is the link to the electronic supplementary material.

Supplementary file1 (PDF 286 KB)

Supplementary file2 (XLSX 207 KB)

## Data Availability

All sequencing data from Guangdong coastal zone are available at National Center for Biotechnology Information (https://www.ncbi.nlm.nih.gov/) under the accession number PRJNA1062668 (amplicon), PRJNA1063027 (metagenome), and PRJNA1063025 (metatranscriptome).
