# Supplementary material for: Community structure and carbon metabolism functions of bacterioplankton in the Guangdong coastal zone
Source: Mar Life Sci Technol. 2024 Jul 29;6(3):547–61. doi: 10.1007/s42995-024-00245-x (PMC11358369; doi:10.1007/s42995-024-00245-x)

Supplementary material for:

Community structure and carbon metabolism functions of bacterioplankton in the Guangdong coastal zone

Fig. S1. Community assembly processes for FL and PA microbial communities. In **(A)**, βNTI distributions for FL and PA microbial community are shown, while **(B)** shows proportions of community assembly processes between FL and PA, respectively. In **(A)**, phylogenetic turnover that is less than null expectations (i.e., βNTI < −2) indicates homogenous selection, phylogenetic turnover that is greater than null expectations (i.e., βNTI > 2) indicates variable selection, and phylogenetic turnover that does not vary from null expectations (|βNTI | < 2) indicates neutral processes. Shades of blue represent selection processes while shades of red represent neutral processes.

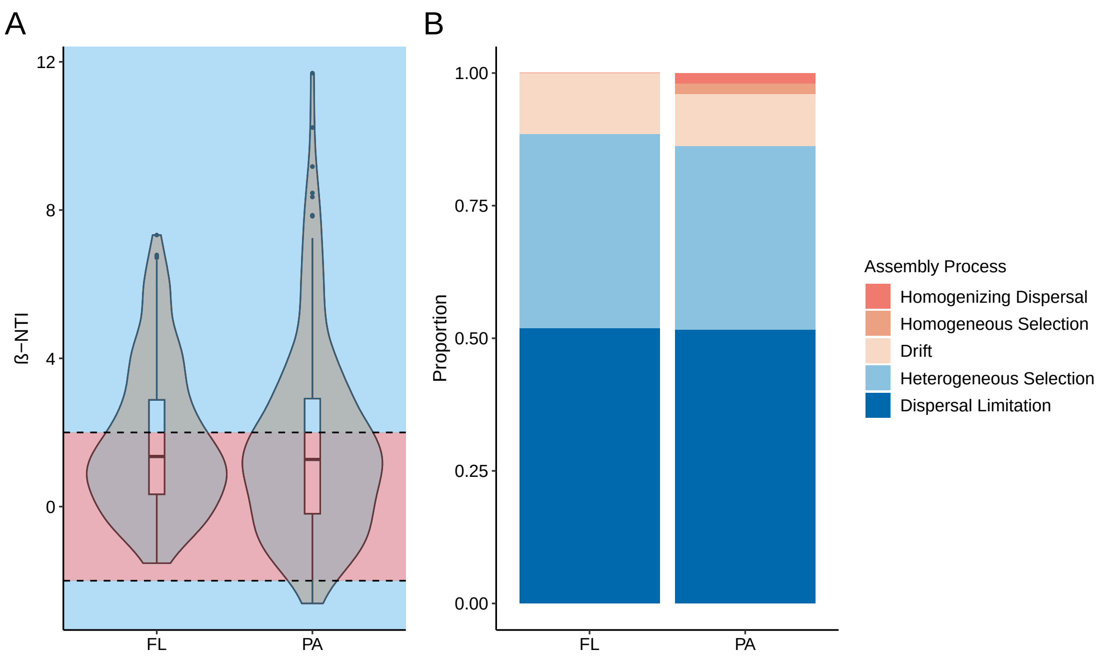

Fig. S2. Carbon degradation (CD) and carbon fixation (CF) gene abundance in the metagenomes (DNA) and metatranscriptomes (RNA) of different lifestyles. (A) The differences of total gene abundance and transcriptional abundance in different lifestyles from an overall aspect. **(B)** The differences of gene abundance in FL and PA in different carbon degradation pathways. **(C)** The differences of gene abundance in FL and PA in different carbon fixation pathways. Wilcoxon rank-sum test was performed to examine whether the difference between FL and PA is significant. The significance levels were denoted as "NS", P>0.05; "*", 0.01<P≤0.05; "**", 0.001<P≤0.01; "***", P≤0.001.

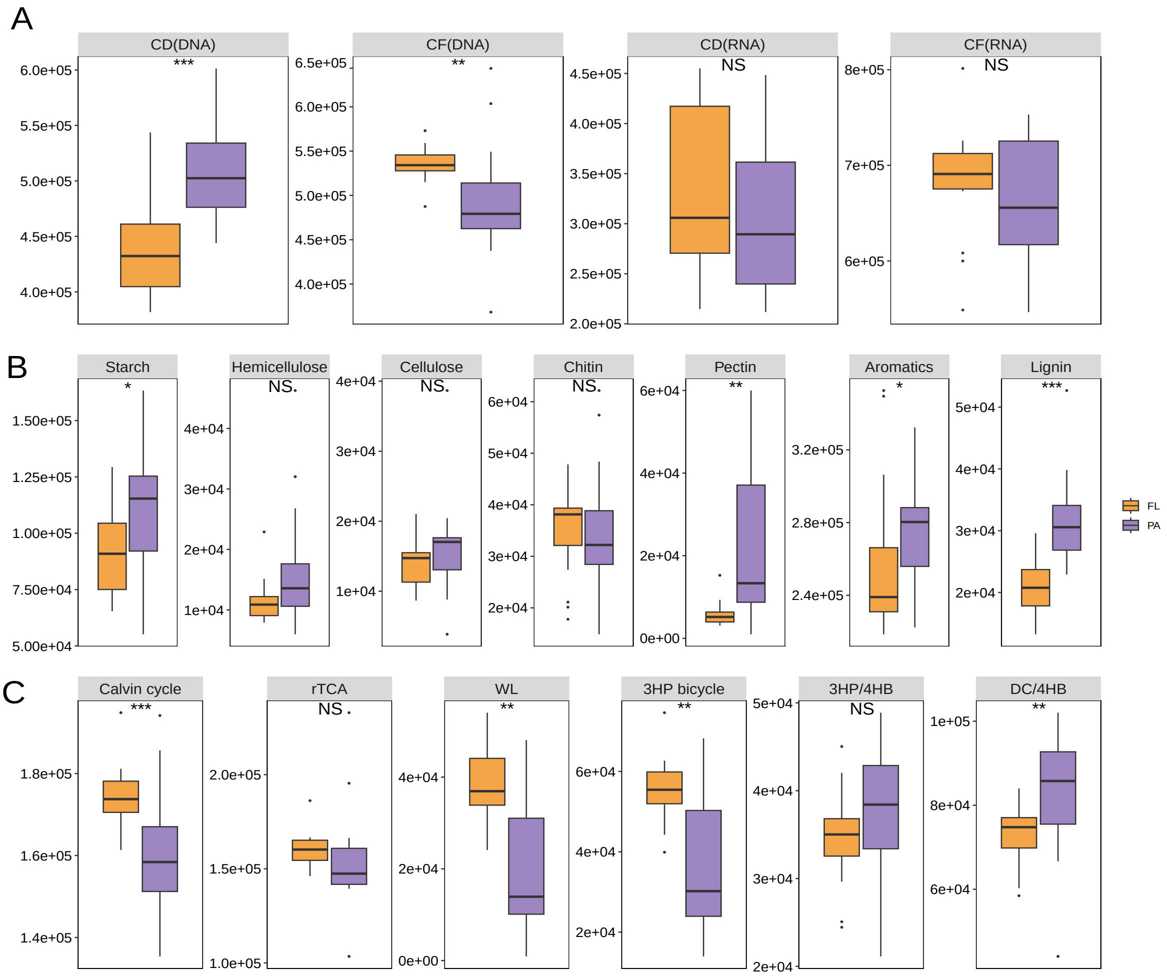

Fig. S3. Class composition abundance maps for each sample community. Top 10 abundant classes were shown and the rest were incorporated and exhibited as “Others”.

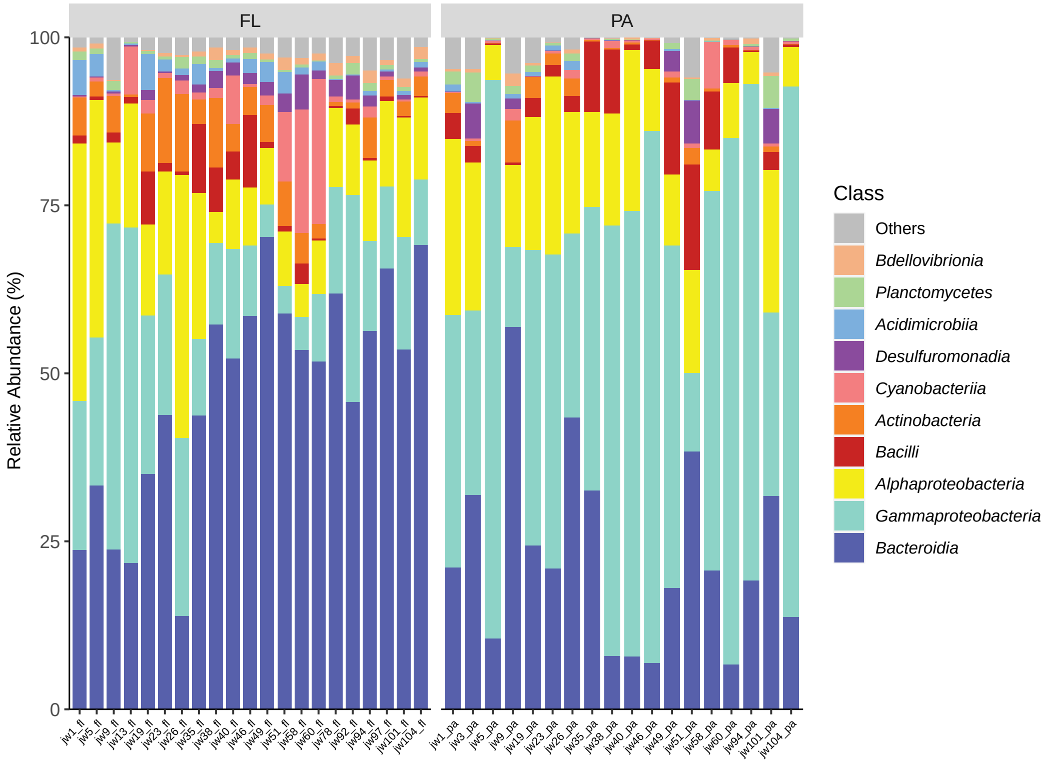

Supplement: Supplementary file 1 — Supplementary file1 (PDF 286 KB) [file 42995_2024_245_MOESM1_ESM.pdf]
